# Comprehensive Analysis of CRISPR/Cas9-Mediated Mutagenesis in *Arabidopsis thaliana* by Genome-Wide Sequencing

**DOI:** 10.3390/ijms20174125

**Published:** 2019-08-23

**Authors:** Wenjie Xu, Wei Fu, Pengyu Zhu, Zhihong Li, Chenguang Wang, Chaonan Wang, Yongjiang Zhang, Shuifang Zhu

**Affiliations:** 1 College of Plant Protection, China Agricultural University, Beijing 100193 China; 2 Institute of Plant Quarantine, Chinese Academy of Inspection and Quarantine, Beijing 100176, China

**Keywords:** *Arabidopsis thaliana*, CRISPR/Cas9, off target, whole-genome sequencing

## Abstract

The clustered regularly interspaced short palindromic repeats (CRISPR)/CRISPR-associated protein (Cas) system has been widely applied in functional genomics research and plant breeding. In contrast to the off-target studies of mammalian cells, there is little evidence for the common occurrence of off-target sites in plants and a great need exists for accurate detection of editing sites. Here, we summarized the precision of CRISPR/Cas9-mediated mutations for 281 targets and found that there is a preference for single nucleotide deletions/insertions and longer deletions starting from 40 nt upstream or ending at 30 nt downstream of the cleavage site, which suggested the candidate sequences for editing sites detection by whole-genome sequencing (WGS). We analyzed the on-/off-target sites of 6 CRISPR/Cas9-mediated *Arabidopsis* plants by the optimized method. The results showed that the on-target editing frequency ranged from 38.1% to 100%, and one off target at a frequency of 9.8%–97.3% cannot be prevented by increasing the specificity or reducing the expression level of the Cas9 enzyme. These results indicated that designing guide RNA with high specificity may be the preferred factor to avoid the off-target events, and it is necessary to predict or detect off-target sites by WGS-based methods for preventing off targets caused by genome differences in different individuals.

## 1. Introduction

The clustered regularly interspaced short palindromic repeats (CRISPR)/CRISPR-associated protein (Cas) system is a bacterial immune system, which combats invading foreign DNA [1,2], and the type II CRISPR/Cas9 system is the most widely used genome editing tool [3,4,5,6,7,8,9,10] because of its simplicity, efficiency, and user-friendliness [11]. CRISPR/Cas9 technology outperforms traditional plant breeding techniques with an unprecedented genome editing precision and efficiency, allowing accurate modification of agronomical traits without any exogenous fragment by genetic segregation. As a new breeding technology, it has been successfully applied in various species [12,13], including blast-resistant rice [14], drought-tolerant maize [15], and browning-resistant mushroom [16]. It provides unprecedented opportunities for molecular breeding applications.

The CRISPR/Cas9 system is composed of a customized single guide RNA (sgRNA) and a Cas9 enzyme, which recognize the target DNA along with the protospacer adjacent motif (PAM, NGG for *Streptococcus pyogenes*) through, approximately, a 20 nucleotide (nt) base-pairing interaction [17]. The double-strand break (DSB) generated by Cas9 activates the nonhomologous end joining (NHEJ) or homology-directed repair (HDR) pathway, causing the knockout or replacement of the target gene. Compared with HDR, the editing efficiency of NHEJ is higher, and it mainly causes random indels of small fragments at the target site. A few studies have found that this system can induce structural variations (SVs) [18]. However, studies have shown that if the PAM is NAG, there is still high editing efficiency [19,20], and sites with up to six base mismatches aligning to the on-target sequence can also be cleaved by the Cas9 of *Streptococcus pyogenes* (SpCas9) [21]. Moreover, single nucleotide variations (SNVs) can also impact the specificity of Cas9. Yang et al. found that a single base genetic variation produced a new off-target site with high editing efficiency in human-induced pluripotent stem cells [22]. Studies of animals showed that off-target activity can be reduced by using improved engineered Cas9 variants [23,24] and controlling the enzyme concentration [25,26,27], but there is little evidence to prove that and few off targets were found in plants [28].

Human-desired variations induced by CRISPR/Cas9 system are not spontaneous variations, and unexpected editing events may be a safety hazard for CRISPR/Cas9-mediated crops [29,30]. Whole-genome sequencing (WGS) has become the preferred method for detecting genome-edited mutations because it provides comprehensive information about genomic variations, including on-/off-target variations [31,32]. Feng et al. and Peterson et al. detected off targets in the T1 and T2 genome-edited mutants of *Arabidopsis* by WGS and found no off-target sites [33,34]. Tang et al. conducted a WGS analysis of 34 plants edited by Cas9 in the T0 and T1 generations, along with three types of control plants in *Oryza sativa* Nipponbare. It was found that the SNVs and indels identified in edited T0 plants are largely background mutations [35]. Similarly, Li et al. demonstrated that the CRISPR/Cas9-edited plants had few off-targets in cotton, which contains a complex and large genome, and the differences between the offspring of the same parent or the number of somatic mutations during tissue culture were much greater than the number of CRISPR/Cas9-induced off-target sites. However, the study also found that 61 potential off-target sites and PAM were generated by genetic variations in wild-type (WT) plants [36]. Designing sgRNA with high specificity by software [37,38,39,40] is the primary method for plant breeders to reduce off-targets.

However, the sequencing depth of WGS is limited, and most of these studies detected off-targets focusing on either predictable off-target sites with a small number of base mismatches or 20–25 nt flanking of these sites, which may lead us to ignore some low-frequency off-targets. This evidence may be insufficient to prove that we can ignore the off-target effect in plants. Actually, many WGS-based methods have been developed to detect or predict off-target sites, including GUIDE-seq [41], Digenome-seq [42], and CIRCLE-seq [21]. These methods have been widely used for off-target detection in animals and results showed that the CRISPR/Cas9 system is highly specific or has varying degrees of off-target effects [23,43]. However, there have been few such applications in plant research, and there is limited evidence for the common occurrence of off-target sites.

Although genome editing in plants is different from gene therapy, mutants without off-target or exogenous fragments can be obtained by backcrossing and selfing, but this process is time consuming and laborious, which will slow down the process in crop breeding. In addition, unpredictable off-target effects attract the attention of the public and regulatory authorities, and there is no unified international regulatory strategies [29]. Therefore, it is necessary to reduce or avoid off-target incidence and accurately examine the molecular characteristics of genome editing plants. Here, we suggested a method for whole-genome detection of editing sites in CRISPR/Cas9-mediated plants by summarizing the precision of on-target editing for more than 10 species. In addition, combining with Digenome-seq and targeted amplification deep sequencing, we examined the influence of specificity and expression level of Cas9 on on-/off-target editing in *Arabidopsis thaliana*.

## 2. Results

### 2.1. Summary of On-Target Variations in CRISPR/Cas9-Edited Plants

In recent years, studies on the detection of gene editing sites by WGS have increased, but there is no clear criterion for identifying small and midsize indels which may be ignored. To capture editing mutations more accurately, we collected the on-target mutations of genome-edited plants in 95 published articles, including more than 10 species, such as *Arabidopsis*, rice, cotton, maize, soybean, and wheat, covering 195 genes and 281 targets. According to the on-target mutations given in the publications, the indel size and the location distribution of all variations were summarized.

**Figure 1:**
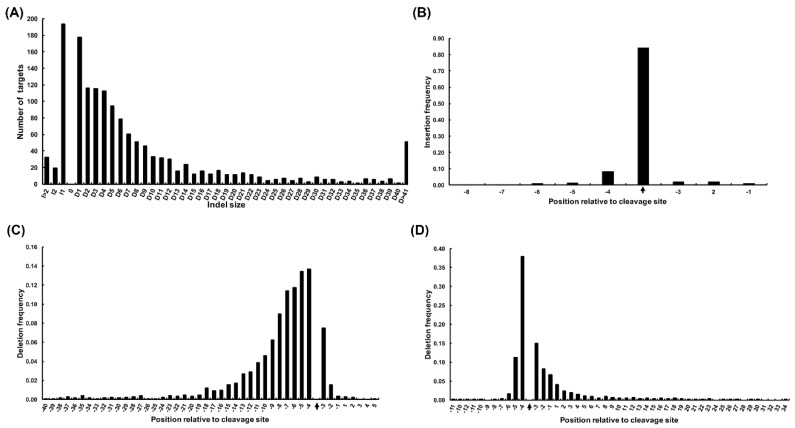
Summary of on-target variations in multiple plants subjected to clustered regularly interspaced short palindromic repeats (CRISPR)/CRISPR-associated protein (Cas9)-mediated editing. (**A**) Distribution of the most common indel size at on-target sites. ‘D’ represents deletion and ‘I’ represents insertion. (**B**) Distribution of the insertion sites of the targets. ‘−1’ on the *X*-axis represents 1 nt upstream of the protospacer adjacent motif (PAM), and black arrows represent cleavage sites. (**C**) Distribution of the starting sites of deletions at target sites. (**D**) Distribution of the ending sites of deletions at target sites.

The analysis results showed that single nucleotide indels were the most common mutations for the majority of targets (Figure 1A). The insertion positions were mainly located at the cleavage sites (commonly 3 nt upstream of PAM) or 1 nt upstream (Figure 1B). Compared to insertions, more targets showed a preference for longer deletions (up to 40 nt), which mainly started from 40 nt upstream to 5 nt downstream of the cleavage site (Figure 1C), and the ending positions extended to 30 nt downstream of the cleavage site (Figure 1D). Based on the results, we determined that the sequences 50 nt upstream and 50 nt downstream of the mutations would be extracted as candidate sequences for editing sites-detection by WGS.

### 2.2. Off-target Sites Predicted by Digenome-seq

zCas9 is a maize codon-optimized SpCas9 mutant with high on-target editing efficiency, but its off-target effect has not been fully evaluated. To detect the DNA targeting specificity of the zCas9 and SpCas9 enzymes in vitro and shorten the number of potential off-target sites, we performed Digenome-seq to capture the cleavage sites of SpCas9 and zCas9. For all 10 target sequences, there were at least 10 base mismatches between each pair of them. The SpCas9/sgRNA^-^ control sample was sequenced to a depth of 45×, and the SpCas9/sgRNA^+^ and zCas9/sgRNA^+^ experimental samples were sequenced to a depth of 70×. The genome mapping ratio of the zCas9/sgRNA^+^ sample was 93.72%, but the SpCas9/sgRNA^−^ sample and the SpCas9/sgRNA^+^ sample were both lower than 80%, which may be caused by random DNA degradation by different enzymes.

**Figure 2:**
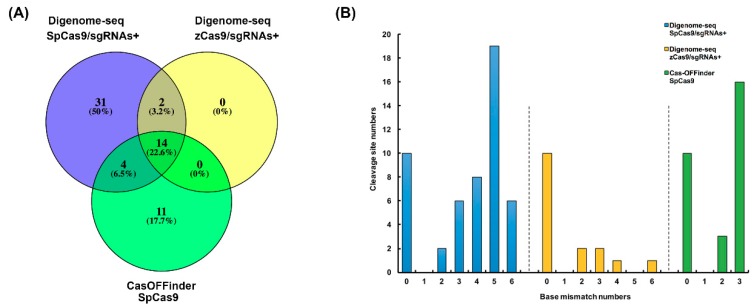
Comparison of off-target prediction results. (**A**) Venn diagram of predicted off-target sites of Digenome-seq and Cas-OFFinder. (**B**) Distribution of base mismatches with on-target sequences of digenome-seq and Cas-OFFinder. The number of mismatches did not include mismatches in the PAM sequence.

Studies have shown that sequences with 6 mismatches mapping to the targets may also be cut by the CRISPR/Cas9 system, so to avoid false positives, we selected potential off-target sites with zero to six mismatches or one bulge with no mismatch. As expected, the SpCas9/sgRNA^−^ control sample did not detect any cleavage sites. SpCas9/sgRNA^+^ and zCas9/sgRNA^+^ samples identified 51 and 16 sites separately, and all off-target sites of zCas9/sgRNA^+^ were included in the SpCas9/sgRNA^+^ result (Figure 2A). Although the samples had different degrees of DNA degradation, the prediction results of Digenome-seq were still credible. These off-targets were classified according to the number of mismatches. There were only four off-targets with three to six mismatches for zCas9/sgRNAs^+^, and the number of cleavage sites was less than SpCas9/sgRNAs^+^, which was 39 sites (Figure 2B). The results indicated that in vitro cleavage specificity of zCas9 was significantly higher than SpCas9. Compared to the prediction results of Cas-OFFinder using the common criteria, which obtained 29 editing sites with zero to three mismatches or one bulge with zero mismatches of the 10 targets, 14 sites were intersected with the Digenome-seq results of zCas9/sgRNAs^+^, and 18 sites intersected with the SpCas9/sgRNAs^+^ results (Figure 2A). We increased the mismatches to five to predict off-targets by the tool (Table 1), and the comparison results showed that predicting off-targets by Digenome-seq effectively reduced the number of potential off-target sites of each target.

### 2.3. Detection of Genome-Wide Variations and Editing Sites

**Figure 3:**
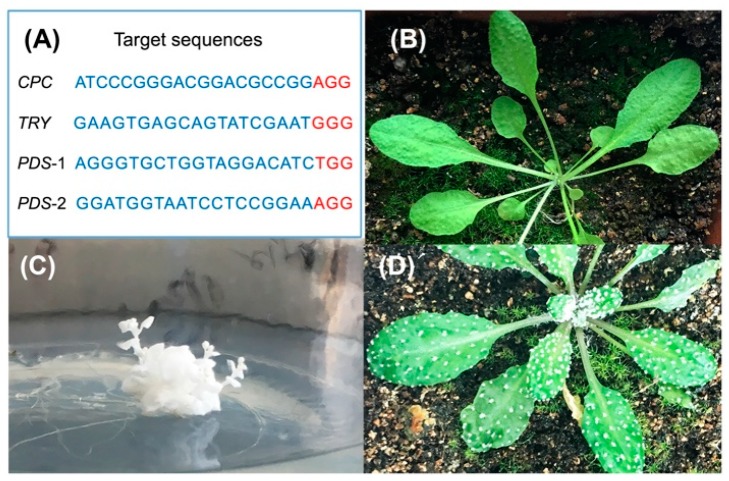
*Arabidopsis* T1 mutants obtained via CRISPR/Cas9. (**A**) Target sequences for constructing CRISPR/Cas9-edited mutants. The 19 nt target sequences and the following PAMs (NGG) are highlighted in blue and red, separately. (**B**) The phenotype of wild-type *Arabidopsis*. (**C**) The phenotype of CRISPR/Cas9-edited *PDS* mutant. (**D**) The phenotype of CRISPR/Cas9-edited *TRY* and *CPC* mutant.

To further study the off-target effects of genome-edited plants, we constructed *TRY*/*CPC* and *PDS*-1/*PDS*-2 double mutants with pHSE401 and pHEE401E expression vectors. *TRY* and *CPC* are two genes that negatively regulate *Arabidopsis* epidermal hair-specific expression genes, and their knockout will cause leaves to form clustered leaf trichomes [44]. The *PDS* gene is a chlorophyll synthesis gene, and the mutants show an albino phenotype [45]. After Sanger sequencing of the antibiotic-screened T1 plants with obvious phenotypes, we obtained *TRY*/*CPC* double mutants with two different vectors (TC/TCE) and *PDS*-2 single mutants with the pHEE401E vector (PDSE) (Figure 3). Six mutants were selected for WGS, including PDSE-3, PDSE-12, TC-15, TC-31, TCE-31, and TCE-42. In addition, three same-generation WT plants of T0 were also included for WGS as control samples to filter spontaneous mutations. All mutants were sequenced with an average of 95× to 140×, and the average sequencing depth of the three WT plants was 80× to 90× (Table S1).

**Figure 4:**
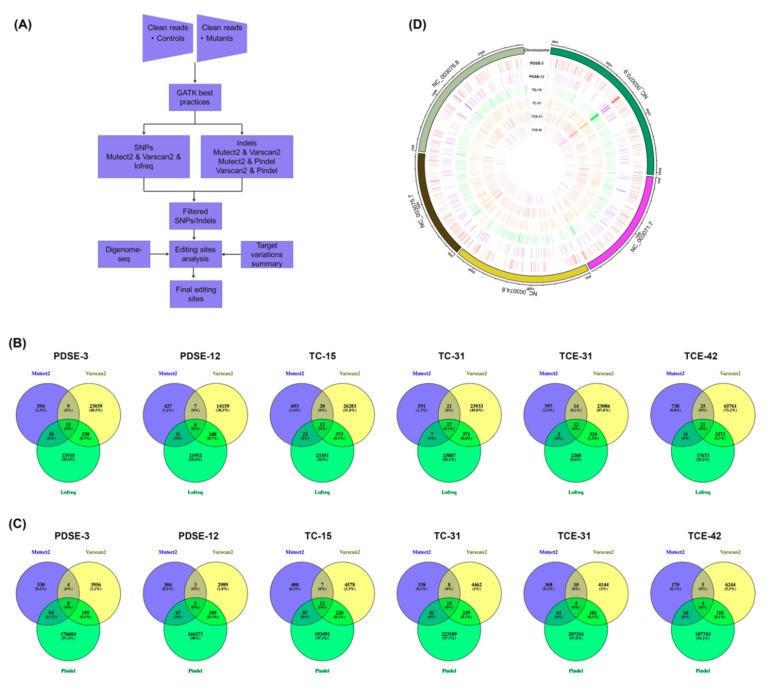
Whole-genome variations analysis in genome-edited mutants. (**A**) Workflow of whole-genome detection of single nucleotide variations (SNVs) and indels. (**B**) Venn diagram of SNVs in genome-edited mutants detected by variant callers. (**C**) Venn diagram of indels in genome-edited mutants detected by variant callers. (**D**) Genome-wide distribution of mutations in CRISPR/Cas9-edited mutants.

First, we identified the T-DNA insertion events. PDSE-3, TC-15, TCE-31, and TCE-42 had only one T-DNA insertion, but both the PDSE-12 and TC-31 mutants had multiple-copy insertions (Figure S1). Then, genome-wide variations were analyzed basing on the pipeline in Figure 4A. To eliminate the influence of the genetic background on the subsequent analysis, the genomic variations of each mutant were subjected to WT variation filtering. There were 608 and 612 SNVs in PDSE-3 and TC-15 mutants, respectively, which were significantly more than other mutants (Table 2). The 573 SNVs of the two mutants were consistent but had no overlap with the SNVs of the other mutants and most of these SNVs were homozygous or heterozygous, which indicated that they may be heritable spontaneous variations from parents. These results suggested that there may be great differences in the genomes of same-generation individuals.

In order to reduce the effects of *Agrobacterium* transfection, different parental genomic differences, sensitivity of software, and variations caused by other factors in each mutant were identified by deducting the variations from mutants with different target sites. The genomic variations of each mutant were significantly reduced, and the number of SNV variants of PDSE-3 and TC-15 were similar to those of the other mutants (Table 2; Figure 4B,C). Further analysis of the genome-wide distribution of these variations in these mutants revealed no hot spots (Figure 4D). For the on-target sites of all mutants, the editing efficiency was 38.1%–100.0% (Table 3). Surprisingly, all *TRY*/*CPC* double mutants had one identical off-target of *TRY*, with two mismatches located in *CPC* gene, and the mutation frequency ranged from 9.8% to 97.3%. The TC-15 mutant had one variation annotated in the repeated sequence with six mismatches of the *TRY* target, which was not predicted by Digenome-seq, so this variation was not a bona fide off-target mutation. We used the same analysis method to reanalyze the published WGS data for the other six sgRNAs in the Digenome-seq and detected all on-target mutations, but no off-target was found. We also detected the complex SVs shorter than 10 kb of these mutants and no SV was found in any editing sites (Table S2).

### 2.4. Analysis of Targeted Amplification Deep Sequencing

Given that leaves are composed of a large number of cells with multiple types, there are various cell types in leaves [34], and multiple editing events may occur in mutants constructed by somatic cell-expressed Cas9. Moreover, low-frequency off-targets may be ignored because of the limited amount of WGS data, so, targeted amplification deep sequencing of PDSE-3; TC-15; TC-31; and pooling samples of WT, PDSE-MIX, TC-MIX, and TCE-MIX were performed to further analyze the editing sites. Except for the PDSE-MIX, which was pooled from 20 mutants, the remaining two samples contained mixtures of five plants. In addition, the zCas9 of TCE mutants was specifically expressed in the egg cell, for which the expression time was shorter than that of TC mutants, and TC mutants with different T-DNA copies had different expression levels of zCas9. Twenty-two sites were subjected to targeted deep sequencing, including all predicted sites of zCas9 by Digenome-seq, off-targets with two to three mismatches predicted by Cas-OFFinder, and randomly selected off-target sites of SpCas9 predicted by Digenome-seq. All sites were sequenced with more than 100,000 reads except the *TRY*-5 site. The on-/off-target mutagenesis frequencies were calculated as the ratio of reads containing indels in the target sites to the total reads captured.

**Figure 5:**
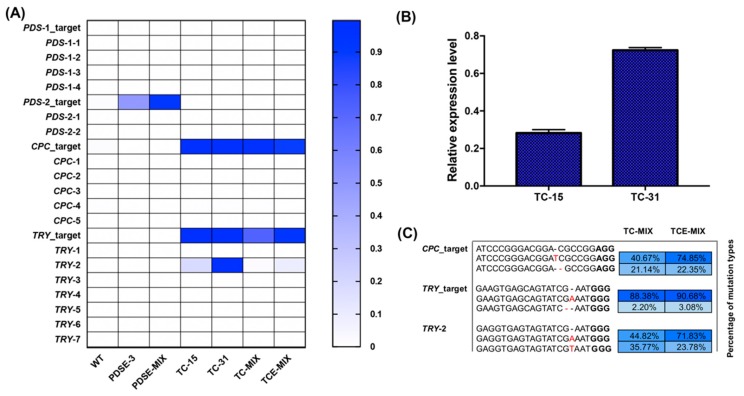
Targeted amplification deep sequencing of genome-edited mutants. (**A**) Heat map of the editing efficiency of on-/off-target sites. (**B**) Comparison of the zCas9 expression of TC-15 and TC-31 by RT-qPCR. The relative expression level was calculated by 2^−ΔCt^ method. (**C**) High proportion of variation types in TC-MIX and TCE-MIX. Deletions and insertions are indicated by “−” and red letters, respectively.

Mutations with frequencies below 1‰ would not be considered bona fide mutations, as such low-frequency mutations may have resulted from sequencing errors. We calculated the on-target editing efficiency via WGS and deep sequencing in PDSE-3, TC-15, and TC-31 and the Pearson correlation between them was 0.997 (p < 0.01). Only one off-target site with two mismatches of the *TRY* target was edited, and the PDS-1 site had no mutations (Table S3; Figure 5A). These results indicated that the deep sequencing results were credible and the whole-genome detection method is effective.

The *CPC* and *TRY* on-target editing efficiency of the TC-31 and TC-15 mutants were above 99.0%, and the off-target efficiency of the TC-31 was equivalent to the on-target sites, which was 98.2%. This result was consistent with the research that off-target editing efficiency may be equal to, or higher than, on-target editing efficiency in human cells [31]. However, the off-target editing efficiency of TC-15 was only 18.6%, indicating that a high expression level of zCas9 may have increased the off-target editing efficiency (Figure 5A,B). Compared with TC-MIX, the editing efficiency of the *TRY* target and the one inevitable off-target in TCE-MIX were relatively high. Moreover, the main mutation types of TC-MIX and TCE-MIX were the same, indicating that repairing via the NHEJ pathway may have some biases (Figure 5C).

## 3. Discussion

In this study, we first collected the target mutations of more than 10 CRISPR/Cas9-mediated species in plants and summarized the size and location distribution of the most common editing mutations. Finally, sequences 50 nt upstream and 50 nt downstream of mutations, detected by WGS, were considered as candidate sequences to identify editing sites by calculating the number of mismatches. The method is different from the currently used detection methods, which focus on the flanking 20–25 nt of the mutations or the potential off-target sites predicted by tools. To verify the effectiveness of the method, we also reanalyzed the public WGS data of four rice mutants [35] by this method and found more off-target sites within three mismatches (Table S4), indicating that the proposed detection method is effective and more sensitive than that used in the publication.

Compared to the large amount of research on CRISPR/Cas9-induced off-targets in animal cells, there are less studies that found off-targets in plants. Although plant researchers have recently tended to use WGS to detect off-target sites instead of using Sanger sequencing, it is difficult to detect low-frequency off-targets because WGS data are limited. Therefore, based on WGS detection of genome editing mutations, we used targeted amplification deep sequencing to further analyze off-targets with mutation frequencies above 1‰. All *TRY*/*CPC* double mutants in this study had one identical off-target, which had two mismatches with the *TRY* target, while the other off-targets with three mismatches and some of the off-target sites predicted by Digenome-seq were not detected. Studies reported that mismatches close to PAM are more critical than mismatches in distal positions [31,46], and Pattanayak et al. suggested that perfect complementarity between sgRNA and target sequences is required in the 7–12 base pairs adjacent to the PAM [47]. Nevertheless, Zhang et al. found that the tolerances to mismatches can occur at any position of the sgRNA [48]. There are differences in the base mismatch complementarity principle between different studies, but the editing efficiency of off-target sites with more than three mismatches is greatly reduced [46,49], so for genome editing plants, most plant breeders focus on off-target sites within three mismatches.

WGS analysis of multiple CRISPR/Cas9 genome-edited mutants of rice showed that the insertion of T-DNA with multiple copy numbers does not induce more off-targets within three mismatches [35]. In this study, the TC-15 and TC-31 mutants had different T-DNA copy number insertions, and there was no difference in the on-target sites. However, the editing efficiency of the off-target site in TC-31 mutants was similar to the on-target efficiency, which was significantly higher than the off-target efficiency in TC-15. This result indicated that a low expression of Cas9 did reduce off-target efficiency, but off-target events could not be completely avoided. Therefore, more attention should be paid to designing sgRNAs with high specificity.

Predicting off-targets by tools is widely accepted by researchers, but different tools have different prediction rules. Some tools predict off-targets by counting the base mismatches and mapping PAM, such as Cas-OFFinder. CHOPCHOP and GT-Scan do not allow mismatches in the seed regions when predicting off-target sites, and some characteristics and parameters can be specified to predict off targets by the Breaking-Cas tool [50]. All tools rely on a known reference genome and only detect predictable off targets. In this study, we found that there were hundreds of SNV differences in different individuals. These heritable spontaneous variations may produce unknown off-target sites that cannot be predicted by software. Studies showed that Cas9 can recognize canonical NGG and noncanonical NAG or NGA PAM sequences. There may still be some sites ignored, even though a large number of off-target sites will be obtained by tools. Although we did not find any targets caused by inherent variations in this study, it is still necessary to comprehensively detect on-/off-targeting by WGS-based methods to assess the safety of genome-edited plants at the molecular level. For species with incomplete genome annotation, de novo sequencing of the genome before designing sgRNA to obtain highly specific target sequences may be required.

In addition, the genome editing mutants constructed in this study used two vectors with *Cauliflower Mosaic Virus* 35S promoter (CaMV 35S) and egg cell-specific promoter, and the deep sequencing results of multiple pooled mutants showed that controlling the expression time of the Cas9 enzyme did not reduce the off-target efficiency. We can avoid complex editing events by selecting appropriate delivery methods for the CRISPR/Cas9 system or developing ‘off-switch’ techniques [51,52] to terminate the expression of the system at a specific time. Shou et al. found that Cas9-mediated nucleotide insertions are nonrandom and are equal to the combined sequences upstream of both PAM sites [53]. Surprisingly, the main mutation types of TC-MIX and TCE-MIX were identical, which suggested that Cas9-mediated nucleotide insertions are nonrandom and there may be some biases in repairing the DSB. The results may be helpful for us to obtain genome-edited plants with specific variations.

In this study, we comprehensively analyzed the variations in on-target sites and the effects of several factors on off-target events in *Arabidopsis*. Compared with nonmodel plants, *Arabidopsis* has a complete genome annotation, and the genome size is only 119.7 Mb (NCBI), which is conducive to clearly revealing the off-target effects of gene editing. Although the number of mutants in this study was limited, the phenomena revealed are useful for the construction of CRISPR/Cas9-edited mutants and the detection of editing sites. To reveal the ubiquitous influence of various factors on CRISPR/Cas9-mediated genome-edited plants, more experimental data may be needed.

## 4. Materials and Methods

### 4.1. Summary of Targeting Precision in Genome-Edited Plants

To collect editing mutations of different target sites and support the detection of off-target variations by WGS, we searched for published CRISPR/Cas9-mediated genome editing articles in PubMed, Science Direct, Elsevier, and other databases with keywords such as “plant”, “crop”, and “CRISPR/Cas9” in the time range of 2014–2019. Then, we analyzed the size and location distribution of mutations caused by CRISPR/Cas9 system. All publications can be found in the Supplementary Materials.

### 4.2. In Vitro Cleavage of Genomic DNA and Cleavage Site Analysis

SpCas9 protein was purchased from New England Biolabs. zCas9 protein was expressed and purified using a previous method [54]. SgRNAs were synthesized using the MEGAshortscript T7 Transcription Kit (Thermo Fisher Scientific, Vilnius, Lithuania) according to the manufacturer’s manual. Transcribed RNA was purified by ethanol precipitation and quantified by spectrometry. SpCas9 or zCas9 protein (40 μg) and 10 sgRNAs (2.7 μg each) were preincubated at room temperature for 10 min to form ribonucleoprotein (RNP) complexes. Genomic DNA (8 μg) was incubated with RNP complexes in NEB reaction buffer 3.1 for 6 h at 37 °C. The digested genomic DNA was treated with RNase A (50 μg/mL) to degrade sgRNAs and purified again with ethanol precipitation. In parallel, genomic DNA without sgRNAs was also processed under the same conditions as a control sample. Then, approximately 1 μg of SpCas9/sgRNA^+^, zCas9/sgRNA^+^, and SpCas9/sgRNA^−^ digested genomic DNA were used for WGS on an Illumina HiSeq X Ten with an average 50× depth at CapitalBio Technology Company (Beijing, China). The identification of sites cut by RNP in vitro was performed as described before [49]. Finally, potential off-targets limited to 6 mismatches mapping to 22 nt on-target sequences were selected for further analysis.

### 4.3. Guide RNA Design and Vector Construction

The sgRNAs were designed according to the CRISPRscan web tool (https://www.crisprscan.org/?page=sequence). The CDS sequences of *PDS, CPC*, and *TRY* target genes were selected for further analysis. First, we found all possible sgRNA sequences in target genes, and for the top-ranked targets, Cas-OFFinder (http://www.rgenome.net/cas-offinder/) was used to predict the off-target sites. Then, two sgRNAs were designed for the *PDS* gene (*PDS*-1 and *PDS*-2) and one sgRNA each for *CPC* (*CPC*) and *TRY* (*TRY*). Plasmids encoding zCas9 and two sgRNAs were generated by ligating pHSE401 [44] or pHEE401E [55] with the pCBC-DT1T2 [44] fragment after digestion with BsaI. All these vectors were provided by Prof. Qijun Chen (China Agricultural University). Moreover, we also selected 6 sgRNAs (*GLV1*-1, *GLV6*-1, *GLV7*-2, *GLV8*-2, *GLV10*-1, and *GLV10*-2) from previous reported research for predicting off-target sites in vitro [34]. All target sequences are listed in Table S5.

### 4.4. Arabidopsis Transformation and Mutagenesis Analysis at On-Target Sites

Two expression vectors were transformed into *Agrobacterium* strain GV3101. WT *Arabidopsis* Col-0 plants were grown in a growth chamber with constant light at 23 °C. Plants were transformed via the floral dip method. Seeds derived from the T0 plants were screened on MS plates containing 25 mg/L hygromycin, and the resistant seedlings were transplanted to soil. Then, the genomic DNA of T1 transgenic plants with obvious phenotypes was extracted with the DNeasy Plant Mini Kit (Qiagen, Hilden, Germany). To confirm mutations in *PDS*, *TRY*, and *CPC*, we amplified fragments surrounding the target sites of the three genes by PCR using specific primers (Table S6).

### 4.5. Whole-Genome Sequencing and Variation Analysis

Genomic DNA was extracted from fresh leaves of three WT plants, two mutants for each CRISPR/Cas9 expression plasmid using the DNeasy Plant Mini Kit. For each sample, approximately 2 μg of DNA was used for WGS on an Illumina HiSeq X Ten (paired-end 150 bp) (CapitalBio Technology Company, Beijing, China), with an average 100× sequencing depth for genomic editing plants and 50× for WT plants at the CapitalBio Technology company. Adapters were trimmed using Cutadapt (v. 1.18) [56]. The WGS data reported in this article are available in the Sequence Read Archive in National Center for Biotechnology (NCBI) BioProject: PRJNA549435.

Cleaned reads were mapped to *Arabidopsis* reference sequence 10.1 (https://www.ncbi.nlm.nih.gov/) with Bowtie2 (v. 2.2.3) [57] software. The Genome Analysis Toolkit (GATK, v. 3.7) [58] was used to realign reads near indels and recalibrate base quality scores by following GATK best practices (https://software.broadinstitute.org/gatk/best-practices/). Whole-genome SNVs were detected by LoFreq (v. 2.1.0) [59], MuTect2 [60], and VarScan2 (v. 2.3.9) [61], and whole-genome indels were identified using MuTect2, VarScan2, and Pindel (v. 0.2.5b9) [62]. Structural variations were analyzed by Delly (v. 0.7.2) [63] and Pindel. To avoid the adverse effects of sequencing errors and missing low-frequency SNVs and indels, common variations detected with at least a depth of 2× for each site were retained for further analysis. After filtering spontaneous variations with WT samples, according to the summary of the common size and location distribution of CRISPR/Cas9-mediated mutations in plants, we captured the upstream and downstream sequences of SNVs that occurred repeatedly in the results from three programs and indels that were found by at least two programs for the editing site analysis. Structural variations within 10 kb detected by both tools were selected for the editing sites identification. Candidate sequences with 0-6 mismatches mapping to 22 nt on-target sequences were compared with the predicted results.

### 4.6. Targeted Amplification Deep Sequencing

Genome-edited and WT plants were used to perform target amplification deep sequencing. Young leaves were used for genomic DNA extraction using the DNeasy Plant Mini Kit. The primers used in the deep sequencing can be found in Table S7. The libraries were purified with a QIAquick PCR Purification Kit (Qiagen, Hilden, Germany) and sequenced on the Illumina X-Ten platform. Bowtie2 was used to map the sequencing data to target sequences, and indels with at least 2× depth were counted as on-target or off-target variations. The ratio of the number of mutant reads to the total number of target reads was used as the editing efficiency.

### 4.7. Real-time Quantitative PCR

Young leaves pooled from 12 T2 plants of TC-15 and TC-31 were used to analyzed the expression levels of the Cas9 by performed the real-time quantitative PCR (RT-qPCR). PCR primers specific to these genes were designed using PrimerQuest Tool (https://sg.idtdna.com/Primerquest/Home/Index) (Table S8). The total RNA was extracted using the RNAprep Pure Plant Kit (Tiangen, Beijing, China) and reverse-transcribed using the protocol of cDNA Synthesis SuperMix (TransGen Biotech, Beijing, China). All RT-qPCR experiments were performed with Applied Biosystems 7900 instruments. The PCR consisted of 10 μL of FastSYBR Mixture (High ROX), 1 μL of forward and reverse primers (10 pM/μL), 7 μL of distilled water, and 1 μL of cDNA in a total volume of 20 μL. The thermal cycling conditions were 3 min at 95 °C followed by 40 reaction cycles (10 s at 95 °C and 20 s at 60 °C). The gene ACTIN2 was used as the reference gene. The mean quantification cycle value of the triple reactions was used to calculate the relative expression levels of the target genes according to the 2^−ΔCt^ method.

## 5. Conclusions

Here, we proposed an accurate method for whole-genome detection of editing sites in CRISPR/Cas9-mediated plants. The results indicated that designing guide RNA with high specificity may be the preferred factor to minimize the possibility of off-targeting, and genome-wide detection of editing sites is inevitable. Our research offered a list of suggestions for how to avoid or reduce the probability of off-target events in plants, which can promote progress in crop improvement by CRISPR/Cas9 system.

## Figures and Tables

**Table 1:** Number of off-target sites predicted by Digenome-seq and Cas-OFFinder.

Nucleotide Mismatch	SgRNAs									
	*GLV* *1*-1	*GLV* *6*-1	*GLV* *7*-2	*GLV* *8*-2	*GLV* *10*-1	*GLV* *10*-2	*PDS*-1	*PDS*-2	*CPC*	*TRY*
0	1/1	1/1	1/1	1/1	1/1	1/1	1/1	1/1	1/1	1/1
1	0/0	0/0	0/0	0/0	0/0	0/0	0/0	0/0	0/0	0/0
2	0/0	0/0	0/0	0/0	0/1	0/0	0/0	0/0	0/0	2/2
3	0/1	0/2	0/3	0/0	1/3	1/5	0/0	0/0	0/0	0/2
4	0/24	0/12	0/52	0/52	0/32	0/33	1/2	0/6	0/37	0/5
5	0/161	0/180	0/350	0/350	0/273	0/273	0/36	0/55	0/27	0/94

Predicted off-target number within 5 mismatches: The number of mismatches did not include mismatches in the PAM sequence. The number in front of the ‘/’ represents the off targets predicted by Digenome-seq and number behind the ‘/’ represent the off targets predicted by Cas-OFFinder tool.

**Table 2:** Variations in CRISPR/Cas9-edited mutants.

Mutant	SNV		Indel	
	WT^−^	WT^−^/DT^-^	WT^−^	WT^−^/DT^-^
PDSE-3	608	15	3120	305
PDSE-12	10	6	1968	184
TC-15	612	12	2525	326
TC-31	31	27	2332	342
TCE-31	14	12	2586	256
TCE-42	24	21	690	143

‘WT^-^’ represents variations in mutants filtered with wild-type (WT) plants; ‘WT^-^/DT^-^’ represents variations in mutants filtered with WT plants and other different target (DT) variations. Variations of CRISPR/Cas9-edited *PDS*, *CPC,* and *TRY* mutants were deducted for each other.

**Table 3:** On-/off-target mutation frequency of WGS.

Mutant	*PDS*-2 (%)	*CPC* (%)	*TRY* (%)	*TRY* Off Target (%)
PDSE-3	38.1	-	-	-
PDSE-12	46.1	-	-	-
TC-15	-	84.1	83.5	20.9
TC-31	-	85.7	88.1	97.3
TCE-31	-	87.6	95.3	9.8
TCE-42	-	100	84.2	19

“-” represents that the site was not the on/off target in the mutant.

## Supplementary Materials

Supplementary materials can be found at https://www.mdpi.com/1422-0067/20/17/4125/s1.

## Abbreviations

CRISPR	Clustered regularly interspaced short palindromic repeats
Cas	CRISPR-associated protein
sgRNA	Single guide RNA
GMOs	Genetically modified organisms
PAM	Protospacer adjacent motif
indel	Insertion and deletion
SNV	Single nucleotide variation
WGS	Whole-genome sequencing
NHEJ	Non-homologous end joining
DSB	Double-strand break
HDR	Homology-directed repair
