# Supplementary material for: Comprehensive Analysis of CRISPR/Cas9-Mediated Mutagenesis in Arabidopsis thaliana by Genome-Wide Sequencing
Source: Int J Mol Sci. 2019 Aug 23;20(17):4125. doi: 10.3390/ijms20174125 (PMC6747142; doi:10.3390/ijms20174125)
Supplement: Supplementary file 1 [file ijms-20-04125-s001.pdf]

Comprehensive Analysis of CRISPR/Cas9-Mediated Mutagenesis in *Arabidopsis thaliana* by Genome-wide Sequencing

Wenjie Xu [1,2], Wei Fu [2], Pengyu Zhu [2], Zhihong Li [1], Chenguang Wang [2], Chaonan Wang [1,2], Yongjiang Zhang [2] and Shuifang Zhu [1,2,*]

[1] College of Plant Protection, China Agricultural University, Beijing 100193 China
[2] Institute of Plant Quarantine, Chinese Academy of Inspection and Quarantine, Beijing 100176, China
[*] Correspondence: zhusf@caiq.org.cn

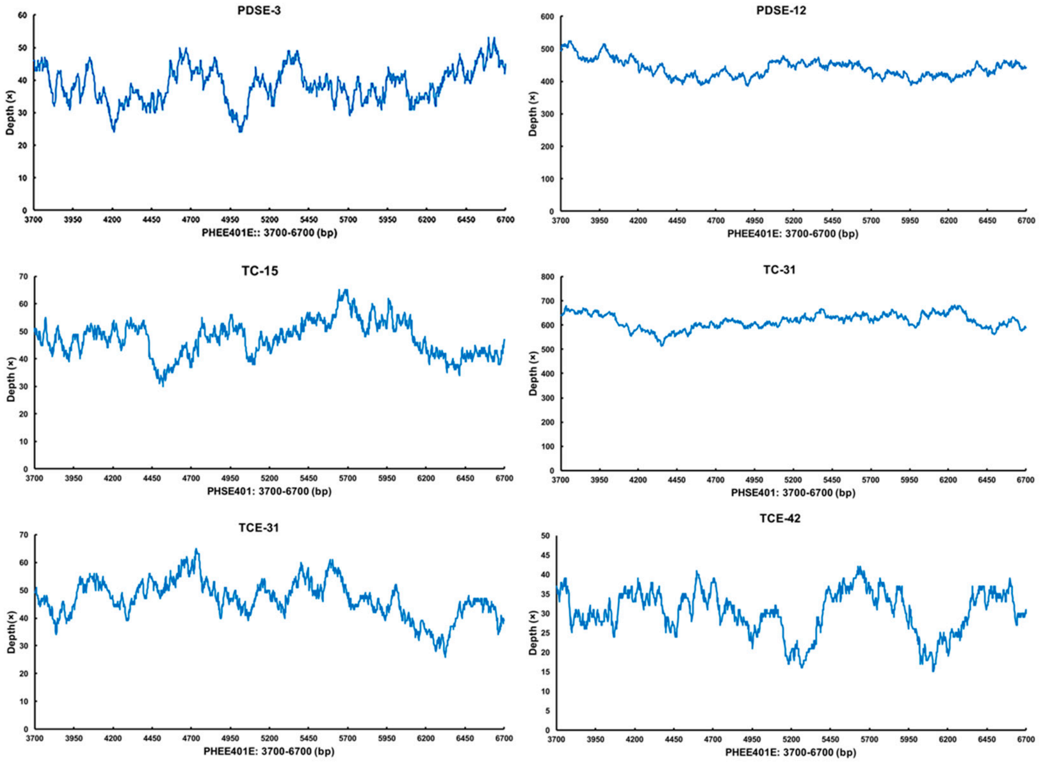

Figure S1. Sequencing depth distribution of inserted vector in the CRISPR/Cas9-edited mutants. The partially continuous specific sequences of two vectors were used for this plot.

Table S1. Sequencing depth and mapping ratio of samples for WGS.

Sample	Sequencing Depth(×)	Mapping Ratio(%)
WT1	94.43	92.61
WT2	84.67	92.86
WT3	87.94	92.74
PDSE-3	139.93	94.36
PDSE-12	132.61	94.43
TC-15	138.52	94.7
TC-31	127.79	94.06
TCE-31	126.64	93.78
TCE-42	95.85	93.67

Table S2. The number of structural variants of mutants.

Mutant	PDSE-12	PDSE-3	TC-15	TC-31	TCE-31	TCE-42
SV	63	72	77	78	80	19
SV（<10kb）	18	16	16	15	24	4

Table S3 Editing efficiency of targets for deep sequencing.

Name	WT	PDSE-3	PDSE-MIX	TC-15	TC-31	TC-MIX	TCE-MIX
PDS-1_target	3.30E-04	2.59E-04	2.21E-04	-	-	-	-
PDS-1-1	5.69E-05	9.10E-05	5.97E-05	-	-	-	-
PDS-1-2	1.74E-04	1.94E-04	1.80E-04	-	-	-	-
PDS-1-3	4.01E-04	4.43E-04	3.32E-04	-	-	-	-
PDS-1-4	1.08E-04	9.09E-05	1.06E-04	-	-	-	-
PDS-2_target	1.28E-02	4.95E-01	9.20E-01	-	-	-	-
PDS-2-1	2.35E-04	2.39E-04	2.08E-04	-	-	-	-
PDS-2-2	7.87E-05	4.94E-05	9.54E-05	-	-	-	-
*CPC*_target	1.41E-02	-	-	9.95E-01	9.95E-01	9.67E-01	8.88E-01
CPC-1	9.64E-05	-	-	1.40E-04	1.55E-04	1.13E-04	1.23E-04
CPC-2	1.08E-04	-	-	1.31E-04	1.15E-04	1.44E-04	1.58E-04
CPC-3	1.62E-04	-	-	1.54E-04	1.39E-04	2.19E-04	1.90E-04
CPC-4	4.66E-04	-	-	6.07E-04	5.92E-04	3.72E-04	3.46E-04
CPC-5	1.68E-04	-	-	1.23E-04	1.12E-04	1.54E-04	1.67E-04
*TRY*_target	2.16E-03	-	-	9.93E-01	9.96E-01	7.24E-01	9.30E-01
TRY-1	1.41E-04	-	-	5.38E-04	2.28E-04	1.85E-04	1.65E-04
TRY-2	2.73E-03	-	-	1.86E-01	9.81E-01	1.61E-02	9.24E-02
TRY-3	2.53E-04	-	-	1.41E-04	1.28E-04	1.71E-04	1.64E-04
TRY-4	4.94E-05	-	-	5.00E-05	3.93E-05	5.20E-05	4.62E-05
TRY-5	1.82E-04	-	-	-	-	-	-
TRY-6	1.40E-04	-	-	2.22E-04	1.61E-04	1.70E-04	1.66E-04
TRY-7	5.40E-04	-	-	5.16E-04	4.82E-04	6.01E-04	5.58E-04

“-” represents that the site in the sample was not detected.

Table S4 Re-analysis results for rice mutants.

Plant Name	NCBI SRA Accession	On Target	Off-target Site	Off-target Sequence
Cas9 Backbone-1	SRR6344862	-	-	-
Cas9 Backbone-2	SRR6344859	-	-	-
Cas9-E1	SRR6344819	Cas9-E	-	-
Cas9-E2	SRR6344879	Cas9-E	-	-
Cas9-J1	SRR6344848	sgRNA01	Chr9:17099504-17099516	GCAGCTCTGACATGTGGGCCCGG
			Chr5:19226195-19226217	ACAGCTCTGACATGTGGGCCCGG
			Chr2:23446231-23446253	GCAGCTCTGACATATGGGCCCGG
			Chr1:31035301-31035323	GCAGCTCTGACATGTGGGTCCGG
			Chr5:13975206-13975228	GGAGCTCTGACATGTGGGCCCGG
			Chr1:22043898-22043920	GCAGCTCTGACATGTGGGCCTGG
			Chr7:22958478-22958500	GCAGCACTGACATGTGGGCCCGG

			Chr9:17967312-17967334	GCAGCTCTGACATGTAGGCCGGG
			Chr12:8340812-8340834	GCAGTTCTGACATGTGGGCCCAG
			Chr10:8570768-8570790	GTAGCTCTGACATATGGGCCGGG
		sgRNA02	-	-
Cas9-J2	SRR6344845	sgRNA01	Chr9:17099504-17099516	GCAGCTCTGACATGTGGGCCCGG
			Chr5:19226195-19226217	ACAGCTCTGACATGTGGGCCCGG
			Chr2:23446231-23446253	GCAGCTCTGACATATGGGCCCGG
			Chr1:31035301-31035323	GCAGCTCTGACATGTGGGTCCGG
			Chr5:13975206-13975228	GGAGCTCTGACATGTGGGCCCGG
			Chr1:22043898-22043920	GCAGCTCTGACATGTGGGCCTGG
			Chr6:24673722-24673744	GCAGCTCTGACATGTGGGCCTGG
			Chr1:19854951-19854973	ACAGCTCCGACATGTGGGCCCGG
			Chr9:17967312-17967334	GCAGCTCTGACATGTAGGCCGGG
			Chr3:2628500-2628522	GCAGCTCTGACATGTGGGTCTGG
			Chr1:4302680-4302702	ATAGCTCTGACATGTGGGCTCGG
			Chr10:8570768-8570790	GTAGCTCTGACATATGGGCCGGG
		sgRNA02	-	-

"-" represents that there is no off target in the mutant for the sgRNA. New off-target sites within 3 mismatches are highlighted in yellow.

Table S5 Target sequences for Digenome-seq.

Target	Sequence
CPC	ATCCCGGGACGGACGCCGGAGG
TRY	GAAGTGAGCAGTATCGAATGGG
PDS-1	AGGGTGCTGGTAGGACATCTGG
PDS-2	GGATGGTAATCCTCCGGAAAGG
GLV1-1	CTTCTTTGGACGTCTTCAATGG
GLV6-1	GCTAGTGCACAAAGAAAGAGGG
GLV7-2	CTGAAACGAAGATGAAGAGAGG
GLV8-2	AAGACAAGCAATAAAGCTGAGG
GLV10-1	AAGGATCATTGAAGCAACATGG
GLV10-2	GATAATCTGCAAATAAGAGCGG

All target sites are listed in 5′ to 3′.

Table S6 Primers used for PCR amplification and sequencing of target sites.

Target	Primer/F	Primer/R
PDS-1	TAAGCAGGATACCAGATGAA	CATGTCAAAGGCGCTAAA
PDS-2	TGTTCTTCCTGACGTCTAA	ACTCATACCCTCTCTGTTG
CPC	CGATGGAGGCTGGTAAA	CTGTCAATGACTGTGTTCAG
TRY	TCTGAAGGTACCTCTCTATTC	GCCAGCCAAGCATTTAT

Table S7 Target sequences and primers used for target amplification deep sequencing.

Name	Target sequence	Primer/F	Primer/R
PDS-1_target	AGGGTGCTGGTAGGACATCTGG	GTTCAACCACTGTTTTCATCAGATAG	GATCAATGATCGGTTGCAGTG
PDS-1-1	GCGCCGCTGGTAGGACATCAGA	GTGCACGCTCAATAGAAACAGC	CAATTACGCTAGTGTTGCCGAC
PDS-1-2	TTGATGCTGGTAGGACAACAGG	GCGAGTTGTTCTCGAGGAGC	CTCTCACAGTTGGGGTGTGCG
PDS-1-3	CTCCTACTGGTTGGACATCTGG	GAGCCCAATGCCACAGGAC	GTGATAATCTTATTGGCTGATTTCG
PDS-1-4	GAGGAGCTGGTAGGACCTTCGG	CGTCACTGGCGCCAGTTC	CATGGCCAAGAAAAATTCCAC
PDS-2_target	GGATGGTAATCCTCCGGAAAGG	GTGCCATCGTCATTGAGCTC	GTGTGTCATAAGCTGCAACAGATG
PDS-2-1	GGATGGTATATCTCTGAAAAGG	CGACGGATACTCAAGTATGGC	GTTTGGGCCGATATTCTTGCTAG
PDS-2-2	TGTTGGATATACTCCGGAATGG	GTTAATTATCAATTTTAAGGTCACACTG	GATTTGGCATTCAATGGAACG
*CPC*_target	ATCCCGGGACGGACGCCGGAGG	CTTGTCTTGTGAATTAAGGAGAGG	GAATAAAAGGTATTTGGTTAAGTGTAAG
CPC-1	CTTCCGAGACAGACGTCGGAGG	GACTGTGATGAATCACCGACAC	GACCAGAGTGATCACGCCAAG
CPC-2	ACTTCAGGACGGACGCTGGAGG	CAAGATCGAAGACGCAATCG	CTTCACCAAATAGTCAAAAGCCTG
CPC-3	ATGGAGGAACCAACGCCGGAGG	GGAGTATTCTGGTCCGAATCAAG	CGCATATATCTCCAGAACAATCTAAG
CPC-4	AAACGGGAACGAACACCGGAGG	GCTGGGGGAAAGACTAAAAAAG	CATGTTTTTCTTATGCGGGGC
CPC-5	TTCCGGCCACGGACGCCGACGG	GTTCACGACCGACGATATCG	GAGTCACCGGTGGAGGAGAAG
*TRY*_target	GAAGTGAGCAGTATCGAATGGG	GATTAGTTTCTAGTTTTAACAAAAATTAATATC	CTTAGAAAAAGTAGAAGAAGAAGAAATTG
TRY-1	GAAGTGAGTAGCATCGAATGGG	GAATCGGACATGACAGAAGAAGAG	CAATTCATTACTCTACACTCATCCTC
TRY-2	GAGGTGAGTAGTATCGAATGGG	GATGGATCGAGAGAGAAAGAGTCTC	GCTTCTTGTTCCGAAGGTCTG
TRY-3	GAAGTGAGCAGTCTTGAGTGGG	CTCCGCTATCTCATGATCTCCATC	GAGAAAACATGAAAAACCTTTCAC
TRY-4	GAAGTGAGTAGTATGAAATGGG	TATATAAACAGAAGAAGAAAAATGTTACG	GTCTACGCTCATTCATTTGACCTG
TRY-5	GAAGTAAGTAGTATCAAAAGGT	GATCACCTAGAAGATAACGGCTATAG	CTGTGGTAAGCTCTACATTCATTGC
TRY-6	GAGATGAAGAGTATCGAATTGG	CTTAATCCATCTTCTCTGATAATCG	GAAGGAGAGTAAAAAGAGACCGTAGAG
TRY-7	GAATACAGCAGTATCAAAGGGT	CGTACTGGCAGATAGAGCAGTTG	CTGATAGGTGCAGCTTTCAAATG

Table S8. Primers used for RT-qPCR amplification.

Gene	Primer/F	Primer/R
ACTIN2	CTGTGCCAATCTACGAGGGTT	CTGTGCCAATCTACGAGGGTT
zCas9	CTACCTGTACTACCTCCAGAAT	GAATGACTGTGGAACGATATGA

All Publications Cited for Section 4.1

1. Amancio, S.; Nakajima, I.; Ban, Y.; Azuma, A.; Onoue, N.; Moriguchi, T.; Yamamoto, T.; Toki, S.; Endo, M. CRISPR/Cas9-mediated targeted mutagenesis in grape. *Plos ONE* **2017,** *12*, e0177966.
2. Andersson, M.; Turesson, H.; Nicolia, A.; Fält, A.S.; Samuelsson, M.; Hofvander, P. Efficient targeted multiallelic mutagenesis in tetraploid potato (Solanum tuberosum) by transient CRISPR-Cas9 expression in protoplasts. *Plant Cell Rep.* **2016**, *36*, 117–128.
3. Andersson, M.; Turesson, H.; Olsson, N.; Fält, A.S.; Ohlsson, P.; Gonzalez, M.N.; Samuelsson, M.; Hofvander, P. Genome editing in potato via CRISPR-Cas9 ribonucleoprotein delivery. *Physiol. Plant* **2018**, *164*, 378–384.
4. Bertier, L.D.; Ron, M.; Huo, H.; Bradford, K.J.; Britt, A.B.; Michelmore, R.W. High-Resolution Analysis of the Efficiency, Heritability, and Editing Outcomes of CRISPR/Cas9-Induced Modifications of NCED4 in Lettuce (Lactuca sativa). *G3 (Bethesda)* **2018**, *8*, 1513–1521.
5. Brooks, C.; Nekrasov, V.; Lippman, Z.B.; Van Eck, J. Efficient Gene Editing in Tomato in the First Generation Using the Clustered Regularly Interspaced Short Palindromic Repeats/CRISPR-Associated9 System. *Plant Physiol.* **2014**, *166*, 1292–1297.
6. Cai, Y.; Chen, L.; Liu, X.; Guo, C.; Sun, S.; Wu, C.; Jiang, B.; Han, T.; Hou, W. CRISPR/Cas9-mediated targeted mutagenesis of GmFT2a delays flowering time in soya bean. *Plant Biotechnol. J.* **2018**, *16*, 176–185.
7. Cai, Y.; Chen, L.; Liu, X.; Sun, S.; Wu, C.; Jiang, B.; Han, T.; Hou, W. CRISPR/Cas9-Mediated Genome Editing in Soybean Hairy Roots. *Plos ONE* **2015**, 10, e0136064.
8. Chandrasekaran, J.; Brumin, M.; Wolf, D.; Leibman, D.; Klap, C.; Pearlsman, M.; Sherman, A.; Arazi, T.; Gal-On, A. Development of broad virus resistance in non-transgenic cucumber using CRISPR/Cas9 technology. *Mol. Plant. Pathol.* **2016**, *17*, 1140–1153.
9. Chen, X.; Lu, X.; Shu, N.; Wang, S.; Wang, J.; Wang, D.; Guo, L.; Ye, W. Targeted mutagenesis in cotton (Gossypium hirsutum L.) using the CRISPR/Cas9 system. *Sci. Rep.* **2017, 7**, 44304.
10. Curtin, S.J.; Xiong, Y.; Michno, J.M.; Campbell, B.W.; Stec, A.O.; Cermak, T.; Starker, C.; Voytas, D.F.; Eamens, A.L.; Stupar, R.M. CRISPR/Cas9 and TALENs generate heritable mutations for genes involved in small RNA processing of Glycine max and Medicago truncatula. *Plant Biotechnol. J.* **2018**, *16*, 1125–1137.
11. D'Ambrosio, C.; Stigliani, A.L.; Giorio, G. CRISPR/Cas9 editing of carotenoid genes in tomato. *Transgenic Res.* **2018**, *27*, 367–378.
12. Deng, L.; Wang, H.; Sun, C.; Li, Q.; Jiang, H.; Du, M.; Li, C.B.; Li, C. Efficient generation of pink-fruited tomatoes using CRISPR/Cas9 system. *J. Genet. Genom.* **2018**, *45*, 51–54.
13. Du, H.; Zeng, X.; Zhao, M.; Cui, X.; Wang, Q.; Yang, H.; Cheng, H.; Yu, D. Efficient targeted mutagenesis in soybean by TALENs and CRISPR/Cas9. *J. Biotechnol.* **2016**, *217*, 90–97.
14. Feng, C.; Su, H.; Bai, H.; Wang, R.; Liu, Y.; Guo, X.; Liu, C.; Zhang, J.; Yuan, J.; Birchler, J.A.; et al. High-efficiency genome editing using a dmc1 promoter-controlled CRISPR/Cas9 system in maize. *Plant Biotechnol. J.* **2018**, *16*, 1848–1857.
15. Feng, C.; Yuan, J.; Wang, R.; Liu, Y.; Birchler, J. A.; Han, F., Efficient Targeted Genome Modification in Maize Using CRISPR/Cas9 System. *J. Genet. Genomics* **2015**, 43, 37-43.
16. Feng, Z.; Zhang, B.; Ding, W.; Liu, X.; Yang, D.L.; Wei, P.; Cao, F.; Zhu, S.; Zhang, F.; Mao, Y.; et al. Efficient genome editing in plants using a CRISPR/Cas system. *Cell Res.* **2013**, *23*, 1229–1232.
17. Gao, R.; Feyissa, B.A.; Croft, M.; Hannoufa, A. Gene editing by CRISPR/Cas9 in the obligatory outcrossing Medicago sativa. *Planta* **2018**, *247*, 1043–1050.
18. Gao, W.; Long, L.; Tian, X.; Xu, F.; Liu, J.; Singh, P.K.; Botella, J.R.; Song, C. Genome Editing in Cotton with the CRISPR/Cas9 System. *Front. Plant Sci.* **2017**, *8*, 1364.
19. Gil-Humanes, J.; Wang, Y.; Liang, Z.; Shan, Q.; Ozuna, C.V.; Sánchez-León, S.; Baltes, N.J.; Starker, C.; Barro, F.; Gao, C.; et al. High-efficiency gene targeting in hexaploid wheat using DNA replicons and CRISPR/Cas9. *Plant J.* **2017**, *89*, 1251–1262.
20. He, Y.; Zhu, M.; Wang, L.; Wu, J.; Wang, Q.; Wang, R.; Zhao, Y. Programmed Self-Elimination of the CRISPR/Cas9 Construct Greatly Accelerates the Isolation of Edited and Transgene-Free Rice Plants. *Mol. Plant* **2018**, *11*, 1210–1213.
21. Ito, Y.; Nishizawa-Yokoi, A.; Endo, M.; Mikami, M.; Toki, S. CRISPR/Cas9-mediated mutagenesis of the RIN locus that regulates tomato fruit ripening. *Biochem. Biophys. Res. Commun.* **2015**, *467*, 76–82.
22. Janga, M.R.; Campbell, L.M.; Rathore, K.S. CRISPR/Cas9-mediated targeted mutagenesis in upland cotton (Gossypium hirsutum L.). *Plant Mol. Biol.* **2017,** *94*, 349–360.

23. Jia, H.; Wang, N. Targeted genome editing of sweet orange using Cas9/sgRNA. *Plos ONE* **2014,** *9*, e93806.
24. Jia, H.; Zhang, Y.; Orbović, V.; Xu, J.; White, F.F.; Jones, J.B.; Wang, N. Genome editing of the disease susceptibility gene CsLOB1 in citrus confers resistance to citrus canker. *Plant Biotechnol. J.* **2017**, *15*, 817–823.
25. Li, J.F.; Norville, J.E.; Aach, J.; McCormack, M.; Zhang, D.; Bush, J.; Church, G.M.; Sheen, J. Multiplex and homologous recombination-mediated genome editing in Arabidopsis and Nicotiana benthamiana using guide RNA and Cas9. *Nat. Biotechnol.* **2013**, 31, 688-691.
26. Jiang, W.; Zhou, H.; Bi, H.; Fromm, M.; Yang, B.; Weeks, D.P. Demonstration of CRISPR/Cas9/sgRNA-mediated targeted gene modification in Arabidopsis, tobacco, sorghum and rice. *Nucleic Acids Res.* **2013,** *41*, e188.
27. Jiang, W.Z.; Henry, I.M.; Lynagh, P.G.; Comai, L.; Cahoon, E.B.; Weeks, D.P. Significant enhancement of fatty acid composition in seeds of the allohexaploid, Camelina sativa, using CRISPR/Cas9 gene editing. *Plant Biotechnol. J.* **2017**, *15*, 648–657.
28. Kapusi, E.; Corcuera-Gomez, M.; Melnik, S.; Stoger, E. Heritable Genomic Fragment Deletions and Small Indels in the Putative ENGase Gene Induced by CRISPR/Cas9 in Barley. *Front. Plant Sci.* **2017**, *8*, 540.
29. Kaur, N.; Alok, A.; Shivani; Kaur, N.; Pandey, P.; Awasthi, P.; Tiwari, S. CRISPR/Cas9-mediated efficient editing in phytoene desaturase (PDS) demonstrates precise manipulation in banana cv. Rasthali genome. *Funct. Integr. Genom.* **2017**, *18*, 89–99.
30. Kim, D.; Alptekin, B.; Budak, H. CRISPR/Cas9 genome editing in wheat. *Funct. Integr. Genom.* **2017**, *18*, 31–41.
31. Klimek-Chodacka, M.; Oleszkiewicz, T.; Lowder, L.G.; Qi, Y.; Baranski, R. Efficient CRISPR/Cas9-based genome editing in carrot cells. *Plant Cell Rep.* **2018**, *37*, 575–586.
32. LeBlanc, C.; Zhang, F.; Mendez, J.; Lozano, Y.; Chatpar, K.; Irish, V.F.; Jacob, Y. Increased efficiency of targeted mutagenesis by CRISPR/Cas9 in plants using heat stress. *Plant J.* **2018**, *93*, 377–386.
33. Lee, K.; Zhang, Y.; Kleinstiver, B.P.; Guo, J.A.; Aryee, M.J.; Miller, J.; Malzahn, A.; Zarecor, S.; Lawrence-Dill, C.J.; Joung, J.K.; et al. Activities and specificities of CRISPR/Cas9 and Cas12a nucleases for targeted mutagenesis in maize. *Plant Biotechnol. J.* **2019**, *17*, 362–372.
34. Li, C.; Chen, C.; Chen, H.; Wang, S.; Chen, X.; Cui, Y. Verification of DNA motifs in Arabidopsis using CRISPR/Cas9-mediated mutagenesis. *Plant Biotechnol. J.* **2018**, *16*, 1446–1451.
35. Li, C.; Hao, M.; Wang, W.; Wang, H.; Chen, F.; Chu, W.; Zhang, B.; Mei, D.; Cheng, H.; Hu, Q. An Efficient CRISPR/Cas9 Platform for Rapidly Generating Simultaneous Mutagenesis of Multiple Gene Homoeologs in Allotetraploid Oilseed Rape. *Front. Plant Sci.* **2018**, *9*, 442.
36. Li, C.; Unver, T.; Zhang, B. A high-efficiency CRISPR/Cas9 system for targeted mutagenesis in Cotton (Gossypium hirsutum L.). *Sci. Rep.* **2017**, *7*, 43902.
37. Li, J.; Manghwar, H.; Sun, L.; Wang, P.; Wang, G.; Sheng, H.; Zhang, J.; Liu, H.; Qin, L.; Rui, H.; et al. Whole genome sequencing reveals rare off-target mutations and considerable inherent genetic or/and somaclonal variations in CRISPR. Cas9-edited cotton plants. *Plant Biotechnol. J.* **2018**, *17*, 858–868.
38. Li, J.; Zhang, H.; Si, X.; Tian, Y.; Chen, K.; Liu, J.; Chen, H.; Gao, C. Generation of thermosensitive male-sterile maize by targeted knockout of the ZmTMS5 gene. *J. Genet. Genom.* **2017**, *44*, 465–468.
39. Li, M.; Li, X.; Zhou, Z.; Wu, P.; Fang, M.; Pan, X.; Lin, Q.; Luo, W.; Wu, G.; Li, H. Reassessment of the Four Yield-related Genes Gn1a, DEP1, GS3, and IPA1 in Rice Using a CRISPR/Cas9 System. *Front. Plant Sci.* **2016,** 7.
40. Li, R.; Fu, D.; Zhu, B.; Luo, Y.; Zhu, H. CRISPR/Cas9-mediated mutagenesis of lncRNA1459 alters tomato fruit ripening. *Plant J.* **2018**, *94*, 513–524.
41. Li, R.; Li, R.; Li, X.; Fu, D.; Zhu, B.; Tian, H.; Luo, Y.; Zhu, H. Multiplexed CRISPR/Cas9-mediated metabolic engineering of γ-aminobutyric acid levels in Solanum lycopersicum. *Plant Biotechnol. J.* **2018**, *16*, 415–427.
42. Li, R.; Zhang, L.; Wang, L.; Chen, L.; Zhao, R.; Sheng, J.; Shen, L. Reduction of Tomato-Plant Chilling Tolerance by CRISPR-Cas9-Mediated SlCBF1 Mutagenesis. *J. Agric. Food Chem.* **2018**, *66*, 9042–9051.
43. Li, S.; Songmei, L.; Yanhua, L.; Haiping, L.; Yuanyuan, T.; Jianzhong, H.; Pengcheng, W.; Yao, S.Q. HRM-facilitated rapid identification and genotyping of mutations induced by CRISPR/Cas9 mutagenesis in rice. *Crop Breed. Appl. Biotechnol.* **2018**, *18*, 184–191.
44. Li, Z.; Liu, Z.B.; Xing, A.; Moon, B.P.; Koellhoffer, J.P.; Huang, L.; Ward, R.T.; Clifton, E.; Falco, S.C.; Cigan, A.M. Cas9-Guide RNA Directed Genome Editing in Soybean. *Plant Physiol.* **2015**, *169*, 960–970.
45. Liang, Z.; Chen, K.; Li, T.; Zhang, Y.; Wang, Y.; Zhao, Q.; Liu, J.; Zhang, H.; Liu, C.; Ran, Y.; et al. Efficient DNA-free genome editing of bread wheat using CRISPR/Cas9 ribonucleoprotein complexes. *Nat. Commun.* **2017**, *8*, 14261.

46. Liang, Z.; Zhang, K.; Chen, K.; Gao, C. Targeted mutagenesis in Zea mays using TALENs and the CRISPR/Cas system. *J. Genet. Genom.* **2014**, *41*, 63–68.
47. Lin, C.S.; Hsu, C.T.; Yang, L.H.; Lee, L.Y.; Fu, J.Y.; Cheng, Q.W.; Wu, F.H.; Hsiao, H.C.; Zhang, Y.; Zhang, R.; et al. Application of protoplast technology to CRISPR/Cas9 mutagenesis: From single-cell mutation detection to mutant plant regeneration. *Plant Biotechnol. J.* **2018**, *16*, 1295–1310.
48. Liu, Y.; Merrick, P.; Zhang, Z.; Ji, C.; Yang, B.; Fei, S.Z. Targeted mutagenesis in tetraploid switchgrass (Panicum virgatum.L.) using CRISPR/Cas9. *Plant Biotechnol. J.* **2018**, *16*, 381–393.
49. Lowder, L.G.; Zhang, D.; Baltes, N.J.; Paul, J.W. 3rd; Tang, X.; Zheng, X.; Voytas, D.F.; Hsieh, T.F.; Zhang, Y.; Qi, Y. A CRISPR/Cas9 Toolbox for Multiplexed Plant Genome Editing and Transcriptional Regulation. *Plant Physiol.* **2015**, *169*, 971–985.
50. Ma, X.; Zhang, Q.; Zhu, Q.; Liu, W.; Chen, Y.; Qiu, R.; Wang, B.; Yang, Z.; Li, H.; Lin, Y.; et al. A Robust CRISPR/Cas9 System for Convenient, High-Efficiency Multiplex Genome Editing in Monocot and Dicot Plants. *Mol. Plant* **2015**, *8*, 1274–1284.
51. Macovei, A.; Sevilla, N.R.; Cantos, C.; Jonson, G.B.; Slamet-Loedin, I.; Cermak, T.; Voytas, D.F.; Choi, I.R.; Chadha-Mohanty, P. Novel alleles of rice eIF4G generated by CRISPR/Cas9-targeted mutagenesis confer resistance to Rice tungro spherical virus. *Plant Biotechnol. J.* **2018**, *16*, 1918–1927.
52. Meng, Y.; Hou, Y.; Wang, H.; Ji, R.; Liu, B.; Wen, J.; Niu, L.; Lin, H. Targeted mutagenesis by CRISPR/Cas9 system in the model legume Medicago truncatula. *Plant. Cell Rep.* **2016**, *36*, 371–374.
53. Nakayasu, M.; Akiyama, R.; Lee, H.J.; Osakabe, K.; Osakabe, Y.; Watanabe, B.; Sugimoto, Y.; Umemoto, N.; Saito, K.; Muranaka, T.; et al. Generation of alpha-solanine-free hairy roots of potato by CRISPR/Cas9 mediated genome editing of the St16DOX gene. *Plant Physiol. Biochem.* **2018**, *131*, 70–77.
54. Nieves-Cordones, M.; Mohamed, S.; Tanoi, K.; Kobayashi, N.I.; Takagi, K.; Vernet, A.; Guiderdoni, E.; Perin, C.; Sentenac, H.; Very, A.A. Production of low-Cs(+) rice plants by inactivation of the K(+) transporter OsHAK1 with the CRISPR-Cas system. *Plant J.* **2017**, *92*, 43–56.
55. Nishitani, C.; Hirai, N.; Komori, S.; Wada, M.; Okada, K.; Osakabe, K.; Yamamoto, T.; Osakabe, Y. Efficient Genome Editing in Apple Using a CRISPR/Cas9 system. *Sci. Rep.* **2016,** *6*, 31481.
56. Odipio, J.; Alicai, T.; Ingelbrecht, I.; Nusinow, D.A.; Bart, R.; Taylor, N.J. Efficient CRISPR/Cas9 Genome Editing of Phytoene desaturase in Cassava. *Front. Plant Sci.* **2017,** *8*, 1780.
57. Osakabe, Y.; Watanabe, T.; Sugano, S.S.; Ueta, R.; Ishihara, R.; Shinozaki, K.; Osakabe, K. Optimization of CRISPR/Cas9 genome editing to modify abiotic stress responses in plants. *Sci. Rep.* **2016**, *6*, 26685.
58. Pan, C.; Ye, L.; Qin, L.; Liu, X.; He, Y.; Wang, J.; Chen, L.; Lu, G. CRISPR/Cas9-mediated efficient and heritable targeted mutagenesis in tomato plants in the first and later generations. *Sci. Rep.* **2016,** *6*, 24765.
59. Ren, C.; Liu, X.; Zhang, Z.; Wang, Y.; Duan, W.; Li, S.; Liang, Z. CRISPR/Cas9-mediated efficient targeted mutagenesis in Chardonnay (Vitis vinifera L.). *Sci. Rep.* **2016**, *6*, 32289.
60. Ron, M.; Kajala, K.; Pauluzzi, G.; Wang, D.; Reynoso, M.A.; Zumstein, K.; Garcha, J.; Winte, S.; Masson, H.; Inagaki, S.; et al. Hairy Root Transformation Using Agrobacterium rhizogenes as a Tool for Exploring Cell Type-Specific Gene Expression and Function Using Tomato as a Model. *Plant. Physiol.* **2014**, *166*, 455–469.
61. Sanchez-Leon, S.; Gil-Humanes, J.; Ozuna, C.V.; Gimenez, M.J.; Sousa, C.; Voytas, D.F.; Barro, F. Low-gluten, nontransgenic wheat engineered with CRISPR/Cas9. *Plant Biotechnol. J.* **2018**, *16*, 902–910.
62. Shan, Q.; Wang, Y.; Li, J.; Zhang, Y.; Chen, K.; Liang, Z.; Zhang, K.; Liu, J.; Xi, J.J.; Qiu, J.L. Targeted genome modification of crop plants using a CRISPR-Cas system. *Nat. Biotechnol.* **2013**, *31*, 686–688.
63. Shen, C.; Que, Z.; Xia, Y.; Tang, N.; Li, D.; He, R.; Cao, M. Knock out of the annexin gene OsAnn3 via CRISPR/Cas9-mediated genome editing decreased cold tolerance in rice. *J. Plant. Biol.* **2017**, *60*, 539–547.
64. Shen, L.; Hua, Y.; Fu, Y.; Li, J.; Liu, Q.; Jiao, X.; Xin, G.; Wang, J.; Wang, X.; Yan, C.; et al. Rapid generation of genetic diversity by multiplex CRISPR/Cas9 genome editing in rice. *Sci. China Life Sci.* **2017**, *60*, 506–515.
65. Shen, L.; Wang, C.; Fu, Y.; Wang, J.; Liu, Q.; Zhang, X.; Yan, C.; Qian, Q.; Wang, K. QTL editing confers opposing yield performance in different rice varieties. *J. Integr. Plant Biol.* **2018,** *60*, 89–93.
66. Singh, M.; Kumar, M.; Albertsen, M.C.; Young, J.K.; Cigan, A.M. Concurrent modifications in the three homeologs of Ms45 gene with CRISPR-Cas9 lead to rapid generation of male sterile bread wheat (Triticum aestivum L.). *Plant Mol. Biol.* **2018,** *97*, 371-383.
67. Soyk, S.; Lemmon, Z.H.; Oved, M.; Fisher, J.; Liberatore, K.L.; Park, S.J.; Goren, A.; Jiang, K.; Ramos, A.; van der Knaap, E.; et al. Bypassing Negative Epistasis on Yield in Tomato Imposed by a Domestication Gene. *Cell* **2017**, *169*, 1142–1155.
68. Soyk, S.; Muller, N.A.; Park, S.J.; Schmalenbach, I.; Jiang, K.; Hayama, R.; Zhang, L.; Van Eck, J.; Jimenez-Gomez, J.M.; Lippman, Z.B. Variation in the flowering gene SELF PRUNING 5G promotes day-neutrality

and early yield in tomato. *Nat. Genet.* **2017**, *49*, 162–168.
69. Sun, Q.; Lin, L.; Liu, D.; Wu, D.; Fang, Y.; Wu, J.; Wang, Y. CRISPR/Cas9-Mediated Multiplex Genome Editing of the BnWRKY11 and BnWRKY70 Genes in Brassica napus L. *Int J. Mol. Sci* **2018,** *19,* E2716.
70. Sun, Y.; Jiao, G.; Liu, Z.; Zhang, X.; Li, J.; Guo, X.; Du, W.; Du, J.; Francis, F.; Zhao, Y.; et al. Generation of High-Amylose Rice through CRISPR/Cas9-Mediated Targeted Mutagenesis of Starch Branching Enzymes. *Front. Plant Sci.* **2017**, *8*, 298.
71. Tang, F.; Yang, S.; Liu, J.; Zhu, H. Rj4, a Gene Controlling Nodulation Specificity in Soybeans, Encodes a Thaumatin-Like Protein But Not the One Previously Reported. *Plant Physiol.* **2016**, *170*, 26–32.
72. Tang, L.; Mao, B.; Li, Y.; Lv, Q.; Zhang, L.; Chen, C.; He, H.; Wang, W.; Zeng, X.; Shao, Y.; et al. Knockout of OsNramp5 using the CRISPR/Cas9 system produces low Cd-accumulating indica rice without compromising yield. *Sci. Rep.* **2017,** 7, 14438.
73. Tang, X.; Zheng, X.; Qi, Y.; Zhang, D.; Cheng, Y.; Tang, A.; Voytas, D.F.; Zhang, Y. A Single Transcript CRISPR-Cas9 System for Efficient Genome Editing in Plants. *Mol. Plant* **2016**, *9*, 1088–1091.
74. Tashkandi, M.; Ali, Z.; Aljedaani, F.; Shami, A.; Mahfouz, M.M. Engineering resistance against Tomato yellow leaf curl virus via the CRISPR/Cas9 system in tomato. *Plant Signal. Behav.* **2018**, 13, e1525996.
75. Tian, S.; Jiang, L.; Gao, Q.; Zhang, J.; Zong, M.; Zhang, H.; Ren, Y.; Guo, S.; Gong, G.; Liu, F.; et al. Efficient CRISPR/Cas9-based gene knockout in watermelon. *Plant Cell Rep.* **2017**, *36*, 399–406.
76. Ueta, R.; Abe, C.; Watanabe, T.; Sugano, S.S.; Ishihara, R.; Ezura, H.; Osakabe, Y.; Osakabe, K. Rapid breeding of parthenocarpic tomato plants using CRISPR/Cas9. *Sci. Rep.* **2017**, *7*, 507.
77. Wang, D.; Samsulrizal, N.H.; Yan, C.; Allcock, N.S.; Craigon, J.; Blanco-Ulate, B.; Ortega-Salazar, I.; Marcus, S.E.; Bagheri, H.M.; Perez Fons, L.; et al. Characterization of CRISPR Mutants Targeting Genes Modulating Pectin Degradation in Ripening Tomato. *Plant Physiol.* **2019**, *179*, 544–557.
78. Wang, F.; Wang, C.; Liu, P.; Lei, C.; Hao, W.; Gao, Y.; Liu, Y.G.; Zhao, K. Enhanced Rice Blast Resistance by CRISPR/Cas9-Targeted Mutagenesis of the ERF Transcription Factor Gene OsERF922. *Plos ONE* **2016,** 11, e0154027.
79. Wang, L.; Chen, L.; Li, R.; Zhao, R.; Yang, M.; Sheng, J.; Shen, L. Reduced Drought Tolerance by CRISPR/Cas9-Mediated SlMAPK3 Mutagenesis in Tomato Plants. *J. Agric. Food Chem.* **2017**, *65*, 8674–8682.
80. Wang, L.; Wang, L.; Tan, Q.; Fan, Q.; Zhu, H.; Hong, Z.; Zhang, Z.; Duanmu, D. Efficient Inactivation of Symbiotic Nitrogen Fixation Related Genes in Lotus japonicus Using CRISPR-Cas9. *Front. Plant Sci.* **2016,** 7.
81. Wang, P.; Zhang, J.; Sun, L.; Ma, Y.; Xu, J.; Liang, S.; Deng, J.; Tan, J.; Zhang, Q.; Tu, L.; et al. High efficient multisites genome editing in allotetraploid cotton (Gossypium hirsutum) using CRISPR/Cas9 system. *Plant Biotechnol. J.* **2018**, *16*, 137–150.
82. Wang, W.; Pan, Q.; He, F.; Akhunova, A.; Chao, S.; Trick, H.; Akhunov, E. Transgenerational CRISPR-Cas9 Activity Facilitates Multiplex Gene Editing in Allopolyploid Wheat. *Cris. J.* **2018**, *1*, 65–74.
83. Wang, X.; Tu, M.; Wang, D.; Liu, J.; Li, Y.; Li, Z.; Wang, Y.; Wang, X. CRISPR/Cas9-mediated efficient targeted mutagenesis in grape in the first generation. *Plant Biotechnol. J.* **2018**, *16*, 844–855.
84. Wang, Y.; Cheng, X.; Shan, Q.; Zhang, Y.; Liu, J.; Gao, C.; Qiu, J.L. Simultaneous editing of three homoeoalleles in hexaploid bread wheat confers heritable resistance to powdery mildew. *Nat. Biotechnol.* **2014**, *32*, 947–951.
85. Wang, Z.; Wang, S.; Li, D.; Zhang, Q.; Li, L.; Zhong, C.; Liu, Y.; Huang, H. Optimized paired-sgRNA/Cas9 cloning and expression cassette triggers high-efficiency multiplex genome editing in kiwifruit. *Plant Biotechnol. J.* **2018**, *16*, 1424–1433.
86. Woo, J.W.; Kim, J.; Kwon, S.I.; Corvalan, C.; Cho, S.W.; Kim, H.; Kim, S.G.; Kim, S.T.; Choe, S.; Kim, J.S. DNA-free genome editing in plants with preassembled CRISPR-Cas9 ribonucleoproteins. *Nat. Biotechnol.* **2015**, *33*, 1162–1164.
87. Xie, K.; Yang, Y. RNA-guided genome editing in plants using a CRISPR-Cas system. *Mol. Plant* **2013**, *6*, 1975–1983.
88. Yang, Y.; Zhu, G.; Li, R.; Yan, S.; Fu, D.; Zhu, B.; Tian, H.; Luo, Y.; Zhu, H. The RNA Editing Factor SlORRM4 Is Required for Normal Fruit Ripening in Tomato. *Plant Physiol.* **2017**, *175*, 1690–1702.
89. Yin, X.; Biswal, A.K.; Dionora, J.; Perdigon, K.M.; Balahadia, C.P.; Mazumdar, S.; Chater, C.; Lin, H.C.; Coe, R.A.; Kretzschmar, T.; et al. CRISPR-Cas9 and CRISPR-Cpf1 mediated targeting of a stomatal developmental gene EPFL9 in rice. *Plant Cell Rep.* **2017**, *36*, 745–757.
90. Yu, Q.H.; Wang, B.; Li, N.; Tang, Y.; Yang, S.; Yang, T.; Xu, J.; Guo, C.; Yan, P.; Wang, Q.; et al. CRISPR/Cas9-induced Targeted Mutagenesis and Gene Replacement to Generate Long-shelf Life Tomato Lines. *Sci. Rep.*

2017, *7*, 11874.
91. Zhang, F.; LeBlanc, C.; Irish, V.F.; Jacob, Y. Rapid and efficient CRISPR/Cas9 gene editing in Citrus using the YAO promoter. *Plant Cell Rep.* **2017**, *36*, 1883–1887.
92. Zhang, J.; Zhang, H.; Botella, J.R.; Zhu, J.K. Generation of new glutinous rice by CRISPR/Cas9-targeted mutagenesis of the Waxy gene in elite rice varieties. *J. Integr. Plant Biol.* **2018**, *60*, 369–375.
93. Zhang, Y.; Li, D.; Zhang, D.; Zhao, X.; Cao, X.; Dong, L.; Liu, J.; Chen, K.; Zhang, H.; Gao, C.; et al. Analysis of the functions of TaGW2 homoeologs in wheat grain weight and protein content traits. *Plant J.* **2018**, *94*, 857–866.
94. Zhang, Y.; Liang, Z.; Zong, Y.; Wang, Y.; Liu, J.; Chen, K.; Qiu, J.-L.; Gao, C. Efficient and transgene-free genome editing in wheat through transient expression of CRISPR/Cas9 DNA or RNA. *Nat. Commun.* **2016,** *7*, 12617.
95. Zhang, Z.; Ge, X.; Luo, X.; Wang, P.; Fan, Q.; Hu, G.; Xiao, J.; Li, F.; Wu, J. Simultaneous Editing of Two Copies of Gh14-3-3d Confers Enhanced Transgene-Clean Plant Defense Against Verticillium dahliae in Allotetraploid Upland Cotton. *Front. Plant Sci.* **2018,** 9, doi: 10.3389/fpls.2018.00842.
